# Assessment of Myocardial Bridge and Mural Coronary Artery Using ECG-Gated 256-Slice CT Angiography: A Retrospective Study

**DOI:** 10.1155/2013/947876

**Published:** 2013-05-20

**Authors:** En-sen Ma, Guo-lin Ma, Hong-wei Yu, Wang Wu, Kefeng Li

**Affiliations:** ^1^Department of Radiology, China-Japan Friendship Hospital, East Yinghua Road, He Pingli, Chao Yang District, Beijing 100029, China; ^2^School of Medicine, University of California, San Diego (UCSD), La Jolla, CA 92121, USA

## Abstract

Recent clinical reports have indicated that myocardial bridge and mural coronary artery complex (MB-MCA) might cause major adverse cardiac events. 256-slice CT angiography (256-slice CTA) is a newly developed CT system with faster scanning and lower radiation dose compared with other CT systems. The objective of this study is to evaluate the morphological features of MB-MCA and determine its changes from diastole to systole phase using 256-slice CTA. The imaging data of 2462 patients were collected retrospectively. Two independent radiologists reviewed the collected images and the diagnosis of MB-MCA was confirmed when consistency was obtained. The length, diameter, and thickness of MB-MCA in diastole and systole phases were recorded, and changes of MB-MCA were calculated. Our results showed that among the 2462 patients examined, 336 have one or multiple MB-MCA (13.6%). Out of 389 MB-MCA segments, 235 sites were located in LAD2 (60.41%). The average diameter change of MCA in LAD2 from systole phase to diastole phase was 1.1 ± 0.4 mm, and 34.9% of MCA have more than 50% diameter stenosis in systole phase. This study suggested that 256-slice CTA multiple-phase reconstruction technique is a reliable method to determine the changes of MB-MCA from diastole to systole phase.

## 1. Introduction

Mural coronary artery (MCA) is defined as the intramural segment of a coronary artery that normally courses epicardially. This condition is also accompanied by a bunch of cardiac muscle fibers that passes over those vessels like a bridge and therefore was called myocardial bridge and mural coronary artery (MB-MCA). It was recognized at autopsy by Reyman in 1737, and its angiographic characteristics were first described by Portmann and Iwig in 1960 [1, 2]. It is considered as an inborn coronary abnormality that most commonly affects the middle third of the left anterior descending artery (LAD) [3]. Traditionally, MB-MCA has been considered a benign condition; however, angina, acute myocardial infarction, ventricular fibrillation, myocardial ischemia, cardiac arrhythmias, and sudden death have been reported in association with MB-MCA [4, 5]. Therefore, the early detection of MB-MCA will help to make clinical intervention in time.

Though conventional angiography (CAG) is considered as gold standard in the diagnosis of MB-MCA, some other new imaging techniques have been used, such as intracoronary Doppler, intravascular ultrasonography (IVUS), and multidetector computed tomography (MDCT). Due to the rapid advancement of CT techniques and equipments, it is technically possible to visualize the vessel wall and plaque and assess the luminal diameter, course, and anatomic relationship of the coronary arteries. Previous studies have described the prevalence, location, morphology, and haemodynamics changes of MB-MCA on the basis of invasive studies such as CAG and IVUS [4–10]. Although recent studies also described the MDCT characteristics of MB-MCA, the haemodynamics changes of MCA during diastole and systole phase were not addressed [11–15]. The objective of this study was to noninvasively evaluate not only the morphological characteristics of MB-MCA but also the changes of MB-MCA from diastole to systole phase with multiple-phase reconstruction technique using 256-slice CT angiography (256-slice CTA).

## 2. Material and Methods

### 2.1. Ethics Statement

The study was approved by our institutional clinical review board as a retrospective investigation. Written informed consent for CT imaging was obtained from all patients and stored in Department of Radiology, China-Japan Friendship Hospital, Beijing, China.

### 2.2. Patients Group

This study was performed between June 2010 and July 2012 and included 2462 consecutive patients (1540 men, 922 women; mean age, 51 ± 4 years; age range, 18–83 years) with atypical chest pain, symptoms suggestive of coronary artery disease (CAD). Each patient had one or more risk factors of CAD such as family history, smoking, hypertension, diabetes mellitus, and hyperlipidemia. Further patient characteristics are given in Table 1. Subjects with history of cardiac surgery, a pacemaker, and arrhythmia were excluded. The average heart rate was 65 ± 11 bpm. Twenty-five to fifty mg of metoprolol was administrated orally one hour before the scheduled CT scan in patients with heart rates higher than 80 beats per minute (bpm) (*n* = 21).

### 2.3. MDCT Scanning Protocol

MDCT scanning of the coronary artery was performed by using a 256-slice MDCT scanner (Philips Brilliance 256; Philips Medical Systems, Cleveland, USA). The entire volume of the heart was covered during one breathhold in approximately 8 to 10 seconds with simultaneous recording of the electrocardiogram (ECG) trace. Further acquisition protocol was as follows: tube voltage, 120 kV; tube current, 420 mAs; detector collimation, 128 × 0.625 mm; increment, 0.625 mm; gantry rotation, 270 ms; field of view, 22–25 cm; matrix size, 512 × 512. 80–100 milliliters of iodinated contrast medium (Ultravist 370, Iopamidol, Schering, Germany) was injected IV at 5 mL/sec followed by 20 mL of saline at 3 mL/sec. As soon as the signal in the ascending aorta reached a predefined threshold of 110 HU, the scan started automatically. Radiation exposure during CT examination was calculated based on the dose length product as reported by Hausleiter et al. [16]. The mean effective dose was calculated as 15.2 to 21.4 mSv for the 256-slice MDCT scanner. 

### 2.4. Data Acquisition and Image Analysis

All CT data were transferred to a commercial available postprocessing workstation (Extended Brilliance Workplace, Philips Medical Systems) with dedicated cardiac analysis software. Both axial resource images and multiplanar reconstructions were evaluated for all patients. Images from all planes for each patient were evaluated no matter whether there is suspicion for myocardial bridging or not. The images were reviewed by two independent radiologists blinded to the patient's history. The diagnosis of MB-MCA was confirmed when consistency was obtained.

**Figure 1:**
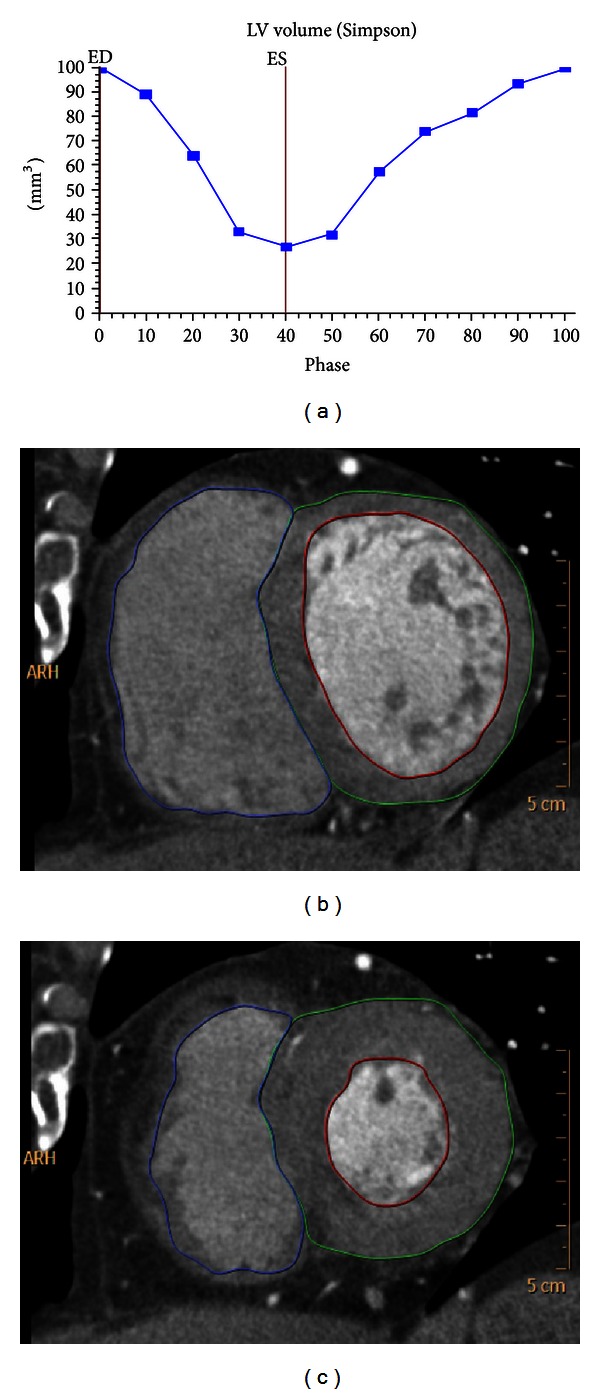
Example of end-systolic (ES) phase determination using left ventricular (LV) volume calculated in a whole cardiac cycle. LV volume-time curve (a), short axis view of LV at ES phase (b), and short axis view of LV at ED phase (c).

The end-systolic (ES) phase and end-diastolic (ED) phase was determined using CT cine technique with cardiac function analysis in patients with MB-MCA. ED phase is the 0 or 0 ms of R-R wave since ECG is accurately synchronized with CT data acquisition [17]. For ES phase selection, we used the way of cardiac function analysis with standard 2.5 mm slice thickness. Ten phases from 0 to 90% with equal 10% R-R interval were reconstructed initially, and the option for semiautomated cardiac function analysis was used for endocardial contour detection and left ventricle (LV) volume calculation. The volume that was derived from the contour drawing in this way was manually adjusted to the level of the mitral valve as we routinely performed in clinical practice. Based on the LV volume-time curves, we could accurately select the ES phase (Figures 1(a), 1(b), and 1(c)).

The length, thickness, and distribution of all MB and atherosclerosis were recorded in mid diastole for optimal motion-free image quality. The length, thickness, and diameter of MB-MCA located at the middle segment of LAD in ED phase and ES phase were recorded at the same level. To avoid motion artifact, near ES phase (30–40% phase) and near ED phase (80–0% phase) were used to demonstrate the MB-MCA in ES and ED phase for measurements; the length, thickness, and diameter of MB-MCA were measured on short axis and sagittal MPR view, then the thickness change (Δthickness) of MB, diameter change (Δdiameter) and length change (Δlength) of MCA in the middle segment of LAD from ED to ES phase were calculated. Each patient was measured at least twice with an interval of two to four weeks for calculating the average by using the same algorithm.

### 2.5. Statistical Analysis

Data analysis was performed using SPSS (version 11.5; Chicago, USA). All data was tested for normal distribution before analysis and the appropriate test was used. All values were expressed as mean ± standard deviation (SD) unless stated in the text. The independent sample *t*-test was used to compare the difference in thickness of MB, diameter and length of MCA between ED and ES phase. A *P* value less than 0.05 was considered as statistically significant difference.

## 3. Results

**Figure 2:**
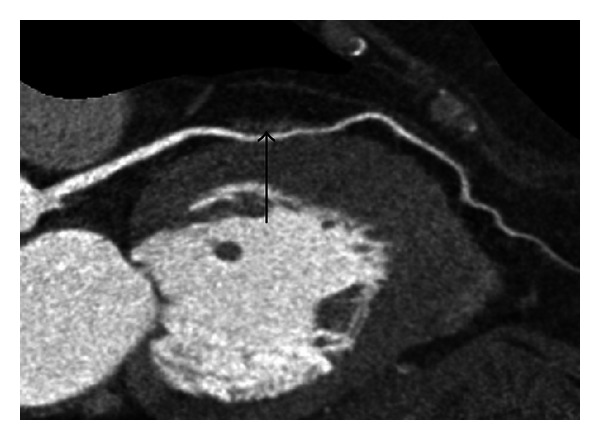
256-slice CT contrast-enhanced coronary angiography images of 51-year-old man with myocardial bridging. Curve multiplanar reconstruction image shows intramyocardial course and shifting into myocardium of middle LAD with typical “step down-step up” phenomenon (arrows). Length of tunneled segment can be clearly measured using the image in ED phase.

Among the 2462 patients, 389 MB-MCA were detected in 336 patients (13.6%). One bridge was found in 297 of these patients, and multiple bridges were observed in 43 patients (33 patients with double bridges and ten with triple bridges). MDCT revealed that MCA shifted into the myocardium with a typical “step down-step up” sign (Figure 2). Fifty-five percent of these patients have chest pain or chest discomfort. Evaluation of the distribution of MB was performed according to the classification of the American Heart Association [18]. 235 MB-MCAs (60.4%) were located in the middle segment of LAD, 59 (15.2%) in the obtuse marginal branch, 53 (13.6%) in the first diagonal artery, and the remaining 42 (10.8%) in the other segments of coronary arteries (Table 2). 

The average length and thickness of MB in all segments of the coronary arteries were 17.6 ± 5.7 mm (ranged from 8 to 40 mm) and 2.7 ± 0.7 mm (ranged from 1.6 to 5.0 mm), respectively.

**Figure 3:**
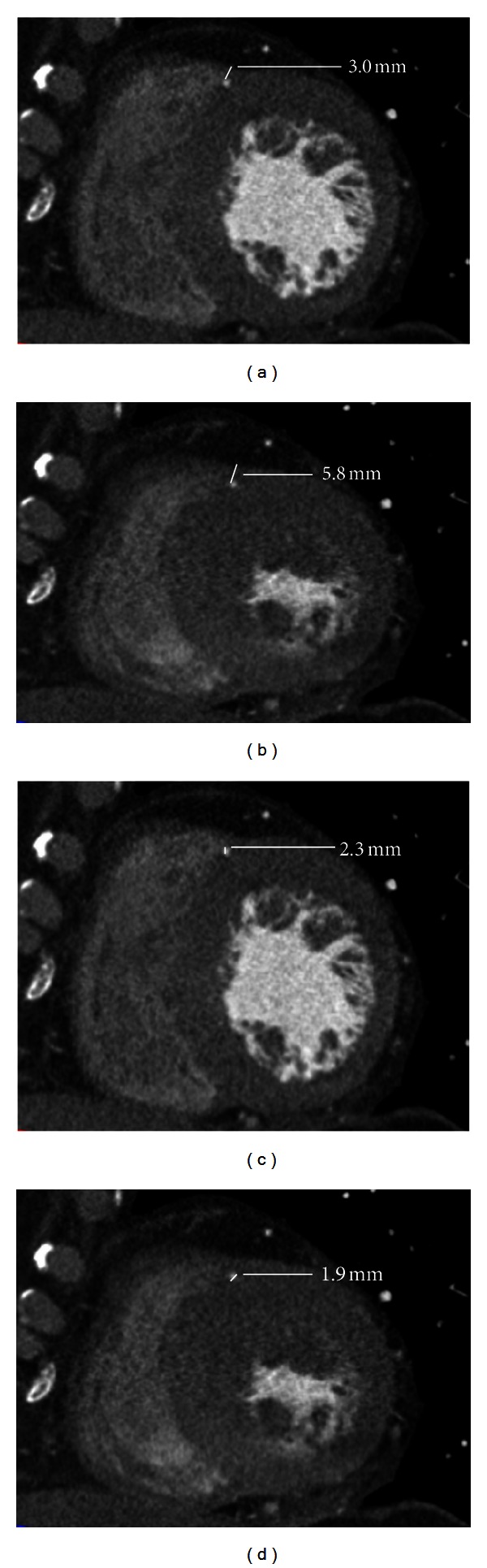
256-slice CT angiography images of 45-year-old man with chest pain for 3 years. Short axis MPR images show LAD encased by a bridge with a thickness of 3.0 mm in ED phase (a) and 5.8 mm in ES phase (b). The diameter of MCA changed from 2.3 mm in ED phase (c) to 1.9 mm in ES phase (d).

**Figure 4:**
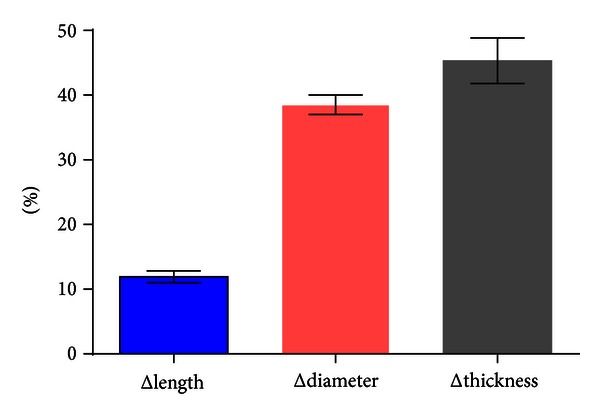
The comparison of the percent change in thickness of MB, diameter and length of MCA from ED to ES phase. The bar graph showed that the percent of thickness change (Δthickness) of MB was the largest from ED to ES phase, followed by the percent of diameter change (Δdiameter) of MCA, and the percent of length change (Δlength) was the least.

Through quantity measurement of MB-MCA that located in the middle segment of LAD, we found that Δthickness of MB, Δdiameter and Δlength of MCA between ED and ES phase were statistically significant (*P* < 0.05, Table 3). The average diameter reduction of MCA in the middle segment of LAD from diastole to systole phase was 1.1 ± 0.4 mm (ranged from 0.3 to 2.3 mm) (Figures 2 and 3).  Figure 4 showed the percent Δthickness of MB, Δdiameter and Δlength of MCA from ED to ES phase, and Δthickness was the largest, followed by Δdiameter, and Δlength was the smallest. Furthermore, 34.9% of the MCA have diameter reduction more than 50% in systole phase.

**Figure 5:**
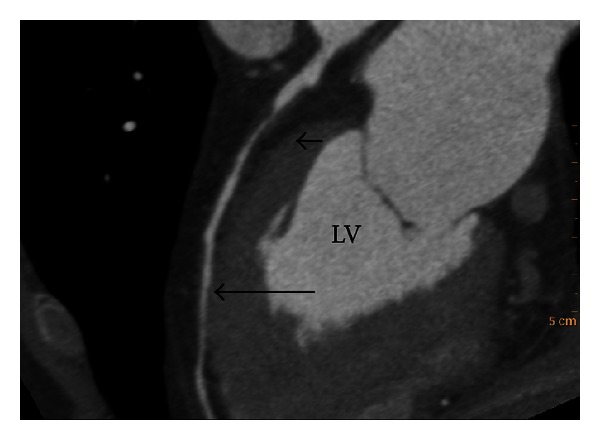
256-slice CT contrast-enhanced coronary angiography image of 44-year-old man with hypercholesterolemia. Curved multiplanar reconstruction image showed long segment of MB on LAD (long arrow) and soft plaque just proximal to bridging (short arrow) in ED phase.

The overall prevalence of atherosclerosis in patients with MB-MCA was 33.4% (112/335), and isolated atherosclerosis plaque was found in 86 patients (26.6%), especially in the proximal segment of MB-MCA (64/335, 19.1%) (Figure 5).

## 4. Discussion

As one of the nonatherosclerotic anatomic abnormalities of the coronary arteries, MB-MCA has become a common disease with the development of coronary artery examination modalities. However, the relationship between MB and symptoms is still unclear. The clinical significance of MB is most likely due to the anatomic narrowing of the tunneled segment caused by the overlying myocardium. Moreover, the presence of concomitant atherosclerosis and stenosis in the coronary artery segment proximal to MB will aggravate myocardial ischemia. Therefore, MB-MCA as a dynamic stenosis, its assessment should not be limited to morphological characteristics but should also include quantification of the diameter reduction from diastole to systole phase.

The current gold standard for diagnosing MB is CAG with the typical “milking effect” and a “step down-step up” phenomenon induced by systolic compression of the tunneled segment [6]. Some other techniques have been used to determine the haemodynamics changes with typical “half moon” phenomenon on IVUS and the early diastolic “finger tip” sign on intracoronary Doppler [13]. However, these techniques are all invasive procedures. As for CAG, MB-MCA can occur in different forms during the systolic and diastolic phases. In addition, interpretation of angiographic findings requires an experienced eye, and only the deep type of bridges may be apparent on angiography. Whereas MDCT could noninvasively delineate the superficial MB and reveal the vessel shifting into the myocardium, and 3D volume-rendering images make it possible for the clinician to see the problem.

The detection rate of MB-MCA in our study was approximately 13.6%. This was higher than previous reports in CAG series (0.5–2.5%) as well as some 16-slice MDCT studies (3.5%), and similar to 256-slice or a dual-source CT study (10.4%), but lower than reports by several pathologic series (15–85%) [11, 19, 20]. These findings indicated that 256-slice MDCT could benefit the diagnosis of MB-MCA due to the increased spatial and temporal resolution. 

The most common coronary artery involved by myocardial bridge is the middle third portions of LAD, whereas the involvement of other coronary arteries, such as RCA, first diagonal, second diagonal, ramus, and the marginal branch, is far less common. In our series, 235 MB-MCAs (60.41%) were located in the middle segment of LAD, then 59 (15.2%) in the obtuse marginal branch, and 53 (13.6%) in the first diagonal artery. This result was in accordance with other studies [3, 15].

As was shown in this study, there was significant difference in the length, thickness, and diameter of 178 segment of MB-MCA in the middle segment of LAD between ED and ES phase. 34.2% of the arteries have diameter reduction more than 50% from diastole to systole phase. This was in accordance with some angiography studies, but the percent of diameter reduction is lower than the previous studies [6–8, 10]. One reason was that we used near systole phase to calculate the diameter reduction for the better image quality, which could not really reflect the diameter in ES phase. Another was maybe that we included more superficial MB, but on angiography studies only deep MB-MCA could be well evaluated. As to the thickness changes of MB, we firstly revealed that it becomes thicker in ES phase, so the MCA was compressed with different extent of diameter reduction and resulted in dynamic stenosis of the coronary artery.

Regarding the occurrence of atherosclerotic plaques in the tunneled coronary segment, some investigators reported that the segments proximal to the MB commonly narrowed, whereas the tunneled segment itself was free of atherosclerotic lesions [21, 22]. This phenomenon has been confirmed by our study, and two mechanisms were suggested for explaining this phenomenon. One was the presence of MB that greatly alters the distribution of the physical forces against the arterial wall. Another was the difference of shear force between the intima beneath the MB and the intima proximal to MB. Thus, alteration of hemodynamic factors may contribute to atherosclerotic plaques formation proximal to the MB and may have a protective role within the MCA [20, 23].

Significance of this study is outlined as follows. First, this study was to investigate such a large patient group (*n* = 2462 patients) and provided the evaluation of the real incidence of myocardial bridging *in vivo*. Next, we evaluated the real changes of MB-MCA from systole to diastole phase using 256-slice MDCT cine technique and found different extent of diameter reduction in ES phase. Finally, the correlation of concomitant atherosclerosis and MB-MCA was also addressed in this study.

Our study found that 256-slice CTA is not suitable for the diagnosis of patients with arrhythmia or patients who cannot hold their breath. Additionally, radiation exposure is, in fact, a major and still debated issue in coronary CTA. Even though the radiation dose in 256-slice CT is substantially lower than 64-slice and dual CTA, optimization for radiation exposure is still required.

## 5. Conclusion 

In summary, the changes of MB-MCA from diastole to systole phase could be determined by 256-slice CTA multiple-phase reconstruction technique. Both the morphological features as well as concomitant atherosclerosis of MB-MCA could be delineated. 256-slice CTA is the optimal modality in the diagnosis of MB-MCA.

## Figures and Tables

**Table 1:** Patients information.

Characteristics	Values
Age (year; mean ± SD)	51 ± 4
Gender (male/female)	1540/922
Typical/atypical chest pain	1034 (42.0)
Chest discomfort	981 (39.9)
Hypertension	340 (13.8)
Diabetes	224 (9.1)
Smoker	467 (19.0)
Family history of CAD	659 (24.9)
Hypercholesterolemia	459 (18.6)

Data in parentheses are percentages; CAD indicates coronary artery disease.

**Table 2:** Classification of MB according to location, thickness, and length of myocardial bridge segment.

Location	No. (%)	Mean length (mm)*	Mean thickness (mm)*
LAD2	235 (60.4)	18.1 ± 6.1	2.9 ± 0.8
OM	59 (15.2)	18.9 ± 4.8	2.4 ± 0.4
D1	53 (13.6)	17.1 ± 4.4	2.7 ± 0.5
D2	14 (3.6)	10.0 ± 2.8	2.1 ± 0.1
LAD1	7 (1.8)	17.0 ± 1.4	2.5 ± 0.7
LAD3	7 (1.8)	13.5 ± 2.1	2.3 ± 0.4
LCX2	7 (1.8)	12.5 ± 0.7	2.2 ± 0.3
RCA2	7 (1.8)	9.0 ± 1.4	3.1 ± 0.1

Total	389 (100)	17.6 ± 5.7	2.7 ± 0.7

Data in parentheses are percentages.

*Data are mean ± standard deviation.

LAD1: the proximal segment of left anterior descending artery (LAD); LAD2: the middle segment of LAD; LAD3: the distal segment of LAD; OM: the obtuse marginal branch; D1: the first diagonal artery; D2: the second diagonal artery; LCX2: the distal segment of left circumflex artery; RCA: the middle segment of right coronary artery.

**Table 3:** Quantity analysis of MB-MCA in the middle segment of LAD (n = 178) during ED and ES phase using CT cine technique.

Quantization analysis	ED*	ES*	Change*	*P* value
Length (mm)	15.5 ± 5.2	17.8 ± 5.6	2.3 ± 1.1	0.018
Thickness (mm)	2.7 ± 0.7	4.0 ± 1.0	1.2 ± 0.4	0.000
Diameter (mm)	2.6 ± 0.5	1.5 ± 0.3	1.1 ± 0.4	0.000

*Data are mean ± standard deviation; ES: end-systolic phase; ED: end-diastolic phase.

Data in parentheses are percentages.
